# iCOD : an integrated clinical omics database based on the systems-pathology view of disease

**DOI:** 10.1186/1471-2164-11-S4-S19

**Published:** 2010-12-02

**Authors:** Kazuro Shimokawa, Kaoru Mogushi, Satoshi Shoji, Atsuko Hiraishi, Keisuke Ido, Hiroshi Mizushima, Hiroshi Tanaka

**Affiliations:** 1 Information Center for Medical Sciences, Tokyo Medical Dental University, Yushima Bunkyo-ku, Tokyo, Japan; 2 Department of Bioinformatics and Computational Biology School of Biomedical Science, Tokyo Medical Dental University, Yushima Bunkyo-ku, Tokyo, Japan

## Abstract

**Background:**

Variety of information relating between genome and the pathological findings in disease will yield a wealth of clues to discover new function, the role of genes and pathways, and future medicine. In addition to molecular information such as gene expression and genome copy number, detailed clinical information is essential for such systematic omics analysis.

**Results:**

In order to provide a basic platform to realize a future medicine based on the integration of molecular and clinico-pathological information of disease, we have developed an integrated clinical omics database (iCOD) in which comprehensive disease information of the patients is collected, including not only molecular omics data such as CGH (Comparative Genomic Hybridization) and gene expression profiles but also comprehensive clinical information such as clinical manifestations, medical images (CT, X-ray, ultrasounds, etc), laboratory tests, drug histories, pathological findings and even life-style/environmental information. The iCOD is developed to combine the molecular and clinico-pathological information of the patients to provide the holistic understanding of the disease. Furthermore, we developed several kinds of integrated view maps of disease in the iCOD, which summarize the comprehensive patient data to provide the information for the interrelation between the molecular omics data and clinico-pathological findings as well as estimation for the disease pathways, such as three layer-linked disease map, disease pathway map, and pathome-genome map.

**Conclusions:**

With these utilities, our iCOD aims to contribute to provide the omics basis of the disease as well as to promote the pathway-directed disease view. The iCOD database is available online, containing 140 patient cases of hepatocellular carcinoma, with raw data of each case as supplemental data set to download. The iCOD and supplemental data can be accessed at

http://omics.tmd.ac.jp/icod_pub_eng

## Background

Recent rapid advances in the human genomics and the subsequent “post-genomic” comprehensive molecular information collectively called “omics” [1,2], such as transcriptome, proteome, metabolome, are bringing about a new possibility of medicine. Such application of molecular information to medicine has been so far called genomic medicine [3], where “personalized medical care” is aimed to be realized based on the inborn individual genomic differences or polymorphisms. Recently, however, post-genomic omics information, for example, gene expression profile (transcriptome) or cellular protein mass spectrometry (proteome) of diseased tissues has been found to be much more directly related to patient’s disease states; it is site-specific in the diseased area and changes through the progression of the disease, so that it can bring about more exact predictive information about the ongoing disease process.

Furthermore, inspired by the rise of the systems biology in the biological science, also in disease study, needs for the systems approach to understand a disease as an integrated whole have been widely recognized. Except for rare monogenetic diseases, most of the diseases can be considered as an integrated system where aberrations of molecular, tissue/organic and individual level are closely interrelated to produce clinical phenotype. We call this perspective “systems pathology” view of disease [4]

With these backgrounds, it becomes accepted that the interrelation between various omics information and clinico-pathological findings of disease is of crucial importance to be clarified in order to develop a new possibility of medicine, which we call “omics-based systems medicine”.

Cancer is now considered as systems dysfunction of cellular regulatory pathway which is caused by the combined effects of environmental/life-style related factor and genetic aberration such as somatic or germline mutations, SNPs, copy number alternation, epigenetic changes and so forth. For diagnosis and therapy of such diseases, not only the molecular information but also clinical, pathological and life-style information is indispensable. Without them, complex diseases such as cancer will not be able to be examined correctly [5-8]. There have been developed many cancer databases [9-11], each of which stores a variety of molecular information. However, more detailed clinical/environmental information in combination with the molecular information is needed to elucidate the whole process of the complex diseases such as cancer. We have first developed an integrated clinical omics database (iCOD), a basic platform where comprehensive disease information of the patient is collected. This database includes not only molecular omics data such as CGH (Comparative Genomic Hybridization) and gene expression profiles but also comprehensive clinical information such as clinical manifestations, medical images (CT, X-ray, ultrasounds, etc), laboratory tests, drug histories, pathological findings and even life-style/environmental information, and gene search menu, related to these clinical information. Furthermore, we developed several kinds of integrated view maps of disease in our iCOD, which summarize the comprehensive patient data to provide the information for the interrelation between the molecular omics data and clinico-pathological findings as well as estimation for the disease pathway, such as three layer-linked disease map, disease pathway map, and pathome-genome map. With these utilities, our iCOD aims to clarify the omics basis of the disease as well as to promote the pathway-directed disease view.

Recently, some pharmaceutical companies announced that they will open the genomic data focus on lung and gastric cancers to rapidly increase knowledge of disease and disease process. So, we can expect that the field of research based on such clinical/environmental information will develop with our iCOD.

### Related work

Cancer Genome Anatomy Project (CGAP) [9]), The Cancer Genome Atlas (TCGA) [10], Cancer Genome Project (CGP) (http://www.sanger.ac.uk/genetics/CGP/) and Atlas of Genetics and Cytogenetics in Oncology and Haematology (AGCOH) [11] are related to our work. However, clinical information is usually only partially treated as Tissue information.

## Construction and content

### Content

**Figure 1:**
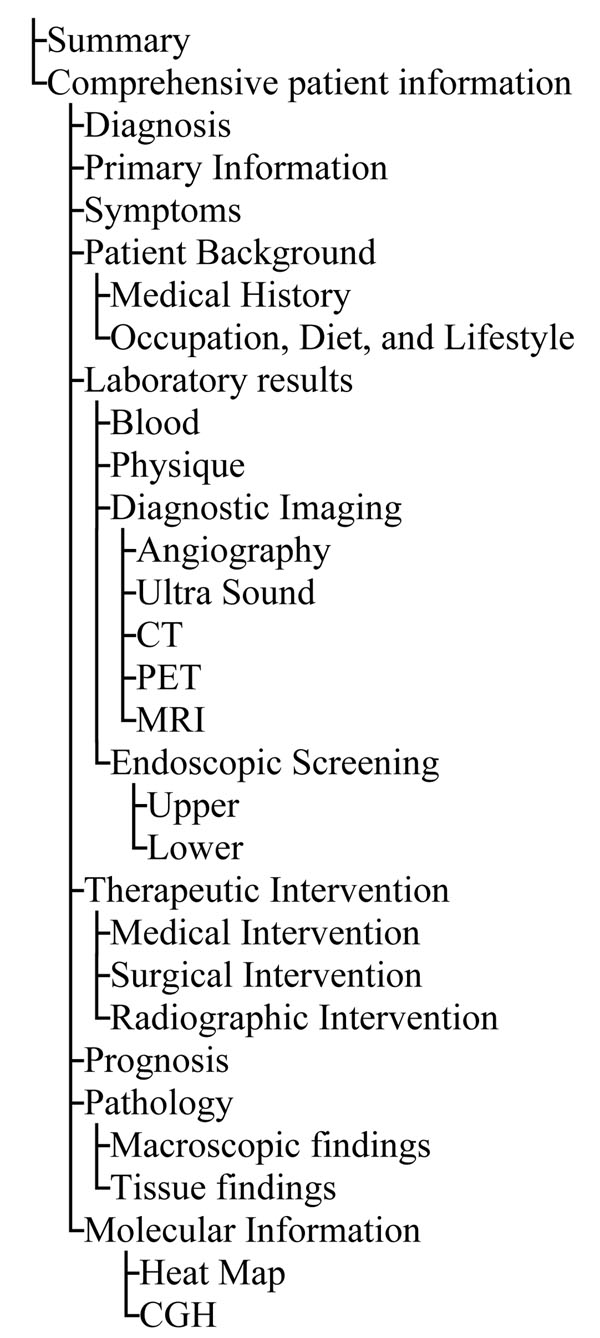
**Collected data items and their hierarchical structure**.

The contents of the iCOD are based on clinical, pathological, and environmental data obtained from patients who received medical care at Tokyo Medical and Dental University Hospital and other collaborating institutions since 2005. At present, we focus on cancer patients. With the tight collaboration with our University hospital, we have collected hepatocellular carcinoma, colon cancer, and oral cancer samples. Samples were collected just after surgery, and were snap-frozen in liquid nitrogen. Meanwhile, clinical research coordinator (CRC) obtained comprehensive clinical records, laboratory data, pathological findings, diagnosis and prognosis, and also performed interview to collect extensive information on medical history, lifestyle and so on. The collected information items are shown in figure 1. A written informed consent was obtained from each patient, and our institutional review board (IRB) approved our iCOD project. Personal information and related items were anonymized and stored in the database.

We also collected gene expression data, array CGH data with detailed pathological information of the sample tissue obtained from each patient. DNA and RNA were extracted from the surgical specimen, after laser capture microdissection which was conducted if required. All of the expression data in the database was obtained using Affymetrix HG-U133 plus 2.0 array as described previously [13]. Array CGH analysis was performed as described in [14]. We have so far collected comprehensive information about several kinds of cancer such as hepatocellular carcinoma, colon and oral cancer of more than 500 cases for its domestic version, of which internationally publicized database is now available online, containing 140 patient cases of hepatocellular carcinoma, which can be browsed at “Case Archive” section in database.

### Implementation

The iCOD was made on the PostgreSQL Database system. This database is capable of storing and handling these clinical/omics data by using 2 dimensional 3 layered (2D-3L) map. The 2D-3L program script is running on the Apache-Tomcat web server.

The back end data analysis programs were written by Java-servlet R statistical software which are available upon request.

## Utility and discussion

**Figure 2:**
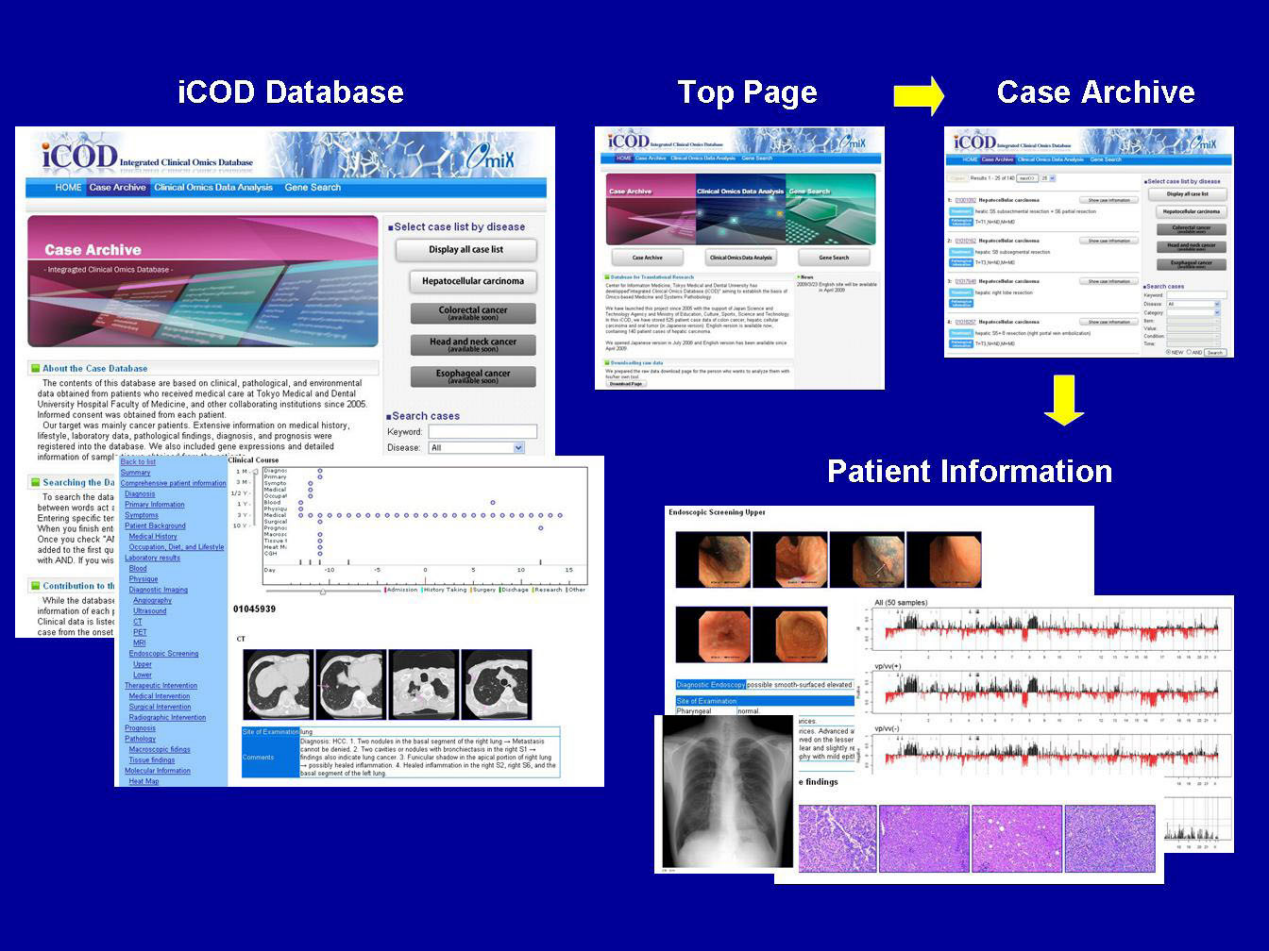
Key screenshot of the iCOD database system.

Figure 2 shows the key screenshot of our iCOD database. iCOD database has two different sections. One is “Case Archive”. The user can browse the patient data by directly viewing the case list or search for the patients having several specific features by use of retrieval function in the section. Another section is “Clinical Omics Data Analysis”. In this section, various kinds of analytical results about the interrelation between clinico-pathological findings and molecular omics or estimation of disease pathways can be seen through the web interface.

### Viewing and searching the case in the database

**Figure 3:**
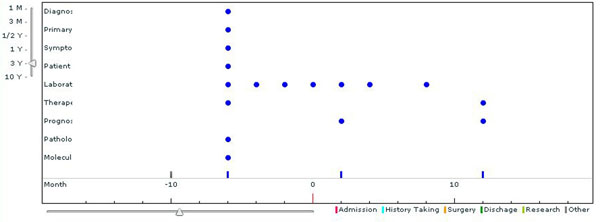
**Time Axis Diagram of data collection history of each patient.** Time Axis Diagram shows time series of medical events of each patient. Each point means date when the data collection was performed. Collected information can be seen by clicking each point in the diagram.

The user can browse the patient list in the database by clicking "Display all case List" in the section of “Case Archive”. To see the details of specific cases, click “Show case information” button. The user will be able to examine further data of an individual patient such as clinical manifestations, medical images (CT, X-ray, ultrasounds, etc), laboratory tests, drug histories, and pathological findings as well as life style information. The case information items and their layered structures are listed in figure 1. The time axis diagram shows the kinds of data stored and their collected dates of each patient in detail (see figure 3).

The iCOD provides users a convenient search engine to query keyword related to pathological/clinical findings and patient ID stored in the database. To search the individual patient cases in the database satisfying the conditions, enter key terms of the query in the “Search” box in the section “Case Archive”.

### Clinical omics data analysis

“Clinical omics data analysis” provides various maps to observe the interrelation (correlation) between clinico-pathological phenotype and gene expression using multivariate statistical analysis applied to the molecular and clinico-pathological information of the patients.

Click on the “Clinical Omics Data Analysis” button from the top page. The user will be able to choose two different analysis methods, which are 2 dimensional 3 layered (2D-3L) map and Pathome-Genome map (CCA).

**Figure 4:**
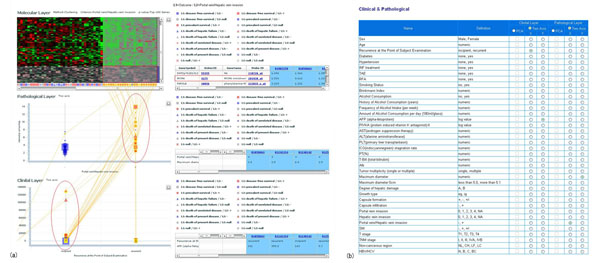
**2 Dimensional 3 Layered map.** Two dimensional three layered map, composed of molecular, pathological and clinical layer map, shows the relative position of the patient within each layer and interrelation between the position of different layer. As shown in this figure, the user can intuitively understand the relation among gene expression pattern (molecular state), pathological state and clinical state of the patient. The axes of the pathological or clinical layer are the first and the second principal component extracted from the principal component analysis applied to the user-specified group of pathological or clinical items, respectively. (a) In the molecular layer, overexpressed genes are chosen, corresponding to the criterion "Portal vein/Hepatic vein invasion +". We can find MCM6 gene, DNA replication licensing factor in the overexpressed genes. On the other hand, pathological layer shows that larger tumors are found (see x-axis of pathological layer) from among this group. Lines between pathological layer and clinical layer show that “incipient” cases (x-axis in clinical layer) usually have larger tumors. (b) Parameter settings, used to draw the map. This system can use the axis specified by the user. In clinical layer, x-axis is recurrence, y-axis is AFP (tumor markers for detection of hepatocellular carcinoma). In pathological layer, x-axis is "Portal vein/Hepatic vein invasion”, y-axis is “Maximum Diameter”.

**Figure 5:**
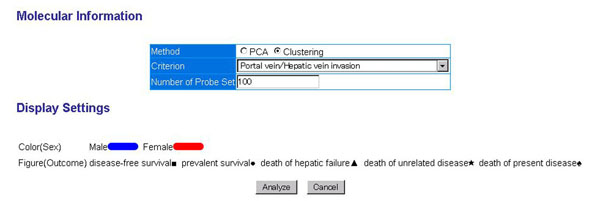
**Gene selection depicted on the heatmap.** The criterion to extract the differentially expressed genes and the number of genes depicted on the heatmap can be specified by the user through the “Parameter setting” page. Genes are extracted by the user-specified criteria of p-value of Wilcoxon rank-sum test.

The 2D-3L map consists of two types of views. The left side view shows the overview of the plot of each patient which provides the relative position of the patient’s information in each of the molecular, pathological and clinical layer. For each layer, principal component analysis (PCA) is used to create 2D map by summarizing the multivariate data into the first and the second principal component scores. The right side view shows the detailed data list in the each layer of the selected patients. Molecular layer displays the result of gene expression profile by a heatmap diagram. In this map, patients are grouped by user-specified criterion selected in the “Parameter Settings” diagram. The screenshot in the figure 4 displays the heatmap in which the criterion of existence or non-existence of “Portal vein/Hepatic vein invasion” is used to extract the differentially expressed genes; the most significant differentially expressed 100 genes are extracted by the user-specified criteria of p-value of Wilcoxon rank-sum test (see figure 5).

In pathological layer and clinical layer, each plot represents patient position in the corresponding 2 principal components coordinate system. By selecting a patient in a certain layer, the 2D-3L map draws connecting line between corresponding points of different layers of the same patient, by which the user can intuitively understand the relationship among different layers of an individual patient. The user can choose multiple patient points at the same time, and the selected patients are shown in the data list; this can be operated by specifying the region including the entire designated patient in the layer with a simple mouse operation.

This map has a parameter setting function for a customized analysis. To use this function, the user only have to change detailed parameter values in three buttons “Data selection”, “Parameter setting” and “Display setting” at the head of the 2D-3L map page. In “Data selection” page, select the type of cancer you wish to analyze (only “Hepatocellular carcinoma” dataset is currently available in the international version with the other cancer datasets in preparation). In “Parameter setting” page, the user will be able to specify the any group of clinical/pathological items to be applied by principle component analysis to determine layer axis.In “Display setting” page, shapes and colours can be adjusted in accordance with various parameters; so that characteristics of a specific group of patients can be obtained by changing these factors for comparison.

Figure 4 shows the case study of the 2D-3L map. First, we can obtain overexpressed or suppressed gene list corresponding to the criterion "Portal vein/Hepatic vein invasion" from the molecular layer. In this case, we found MCM6 gene, DNA replication licensing factor. We are also able to confirm the relation between patient's recurrence and the size of tumor, corresponding to the above-mentioned criterion. Please look at the explanation of figure 4.

**Figure 6:**
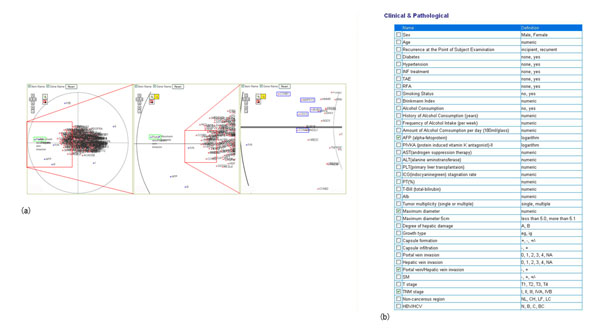
**Pathome-Genome map.** The result of the canonical correlation analysis applied to clinico-pathological findings and gene expression profile. By using this map, the relation between genes and clinical/pathological findings is easily understood. (a) Genes and clinico-pathological findings at the closer position in the map mean that they have higher correlation each other. The enlarged view shows that upregulation of cell cycle-associated genes such as cyclin family CCNA2/B1, MAPK13, BUB, and CDC2, enclosed by blue rectangles, significantly correlated with Portal vein/Hepatic vain invasion, enclosed by green rectangles. This factor is directly associated with well-established clinical criteria such as TNM stage (IVA). (b) Parameter settings, used to draw the map.

Pathome-Genome map shows the relation between clinical/pathological information and gene expression, which was calculated by the regularized canonical correlation analysis (CCA) method (figure 6(a)). CCA is a generalized version of multiple regression analysis, and the associations between two groups of variables are obtained by maximizing the correlation coefficient between the linear combination of each group of variables. In Pathome-Genome map, CCA is used to analyze and visualize the correlation structure between clinico-pathological factors and genes. So, the user can understand the interrelation between two different kinds of data in a same two dimensional coordinates. As described in previous paragraph, the user can arbitrary select the type of cancer and specify the clinical/pathological items he wish to analyze. In this case, we examined what genes are related for a certain clinical items (AFP, Maximum Diameter, Portal vein/Hepatic vain invasion, and TNM). Figure 6(a) clearly shows the relation between Portal vein/Hepatic vain invasion and cell cycle related genes (CCNA2/B1, MAPK13, BUB, and CDC2).

### Future development

Our international version is available now, containing 140 patient cases of hepatocellular carcinoma. The number of cases are increasing and containing the other disease cases such as colon and oral cancer. We also plan to prepare retrieval page that displays the correspondence table of arbitrary gene and its p-values of all criterions used in this data base. We are preparing to accept clinical omics data from other public projects as a repository. We are also preparing to disclose our web based analysis tool for microarray called “Microarray Analysis Workflow”, used to build our database.

## Conclusions

Many cancer related databases which stored a variety of molecular information have been developed, as described before. However, more detailed clinical/environmental information in combination with the molecular information is needed to elucidate the whole process of the complex diseases such as cancer. From this point of view, our iCOD is the first database which provides the comprehensive clinical, pathological and life-style information in addition to the molecular biological information as well as their estimated interrelation. The iCOD database is useful both for clinical researchers who intend to have knowledge about molecular basis of disease which could be used for diagnosis, therapy and prognosis of the diseases, and for molecular biologists who intend to know the function and phenotype of the molecular pathways and their interrelation through the knowledge in the cases of their dysfunction. Our subproject aims to develop the model disease database in the “omics” era which has a standardized database organization being able to cover the multi-hierarchical (from molecular to clinical level) information concerning the diseases.

## Availability and requirements

We prepared the download page of raw gene expression data for users who want to analyze them with his/her own tool. The supplemental data can be found at http://omics.tmd.ac.jp/icod_pub_eng/download. Raw data files consist of raw gene expression data by Affymetrix .CEL binary format, and detailed clinical information of each case is stored by CSV text format.

## Authors’ contributions

KS drafted the manuscript. KS is responsible for achievement and the organization of the web design. KM calculated all p-values and canonical correlation analysis concerning hepatocellular carcinoma. SS and AH checked clinical information, and translated them into English. HM organized the molecular biology experiment. HT and HM provided advice and supervised the research group. All authors read and approved the final manuscript.

## Competing interests

The authors declare that they have no competing interests.
